# Possibilities and limitations of digital microscopy of blood smear of the modern hematological analyser Sysmex XN-3100 in leukocyte differentiation

**DOI:** 10.5937/jomb0-55966

**Published:** 2025-08-21

**Authors:** Nermina Klapuh-Bukvić, Zehra Kurtanović, Damir Šeper

**Affiliations:** 1 Clinical Center University of Sarajevo, Sarajevo, B&H; 2 University of Sarajevo, Faculty of Health Studies, Sarajevo, B&H; 3 University of Sarajevo, Faculty of Pharmacy, Sarajevo, B&H; 4 Public Institution Health Center of Sarajevo Canton, Sarajevo, B&H; 5 Public Institution General Hospital "Prim. dr. Abdulah Nakas" Sarajevo, B&H

**Keywords:** digital microscopy, leukocyte differentiation, Sysmex XN-3100, digitalna mikroskopija, diferencijacija leukocita, Sysmex XN-3100

## Abstract

**Background:**

Differentiation of leukocytes is one of the key diagnostic procedures in clinical medicine, and correct identification of them in a blood smear is of essential importance. Light microscopy is the reference method for leukocyte differentiation; however, it is time-consuming and must be performed by a highly qualified specialist. For this reason, automatic analysers capable of precise and accurate differentiation of blood cells in the examined sample are increasingly present in haematology laboratories. This paper aims to evaluate the performance of the Sysmex XN-3100 analyser, manufactured by SYSMEX CORPORATION, Kobe, Japan., with a focus on the advantages and disadvantages of its digital microscopy in the differentiation of leukocytes and give brief guidelines on the possibilities and limitations of everyday work on the basis of the obtained results.

**Methods:**

Digital optical microscopy on 253 samples was performed with primary data (preclassification) collected after the completion of the autoanalysis. Before validating the obtained results, the data were reviewed by a medical biochemistry specialist who confirmed or corrected them. This generated secondary data (reclassification). The two groups of data were statistically analysed using Passing-Bablok regression analysis, Bland-Altman analysis and Spearman correlation.

**Results:**

The obtained results showed strong correlations between the primary and secondary analysis in all cells (highest in lymphocyte group (r=0.986), lowest in eosinophil group (r=0.870)) except immature granulocytes and blasts (significant deviation from linearity, p<0.01).

**Conclusions:**

The haematology analyser Sysmex XN-3100 shows high performance in leukocyte analysis and differentiation using digital microscopy, but samples containing blasts and immature granulocytes must additionally be analysed by light microscopy.

## Abstract (sr)

**Uvod:**

Diferencijacija leukocita predstavlja jednu od ključnih dijagnostičkih procedura u kliničkoj medicini, a njihova pravilna identifikacija u razmazu krvi je od suštinske važnosti. Svetlosna mikroskopija je referentna metoda za diferencijaciju leukocita, ali je vremenski zahtevna i mora da je obavlja visoko kvalifikovan stručnjak. Zbog toga su automatski analizatori, sposobni za preciznu i tačnu diferencijaciju ćelija krvi u ispitivanom uzorku, sve prisutniji u hematološkim laboratorijama. Cilj ovog rada je da se oceni efikasnost analizatora Sysmex XN-3100, proizvođača SYSMEX CORPORATION, Kobe, Japan, sa posebnim osvrtom na prednosti i nedostatke njegove digitalne mikroskopije u diferencijaciji leukocita, te da se, na osnovu dobijenih rezultata, daju kratke smernice o mogućnostima i ograničenjima njegove primene u svakodnevnom radu.

**Metode:**

Digitalna optička mikroskopija primenjena je na 253 uzorka, pri čemu su primarni podaci (preklasifikacija) prikupljeni nakon završetka autoanalize. Pre potvrđivanja dobijenih rezultata, podatke je pregledao specijalista medicinske biohemije, koji ih je potvrdio ili korigovao. Tako su dobijeni sekundarni podaci (reklasifikacija). Obe grupe podataka statistički su analizirane pomoću Passing-Bablok regresione analize, Bland-Altman analize i Spearmanove korelacije.

**Rezultati:**

Dobijeni rezultati ukazuju na jake korelacije između primarne i sekundarne analize kod svih ćelija (najviša u grupi limfocita (r=0,986), najniža u grupi eozinofila (r=0,870)), osim kod nezrelih granulocita i blasta (značajno odstupanje od linearnosti, p<0,01).

**Zaključak:**

Hematološki analizator Sysmex XN-3100 pokazuje visok nivo efikasnosti u analizi i diferencijaciji leukocita pomoću digitalne mikroskopije, ali uzorke koji sadrže blaste i nezrele granulocite je neophodno dodatno analizirati svetlosnom mikroskopijom.

## Introduction

Leukocytes are white blood cells produced in the bone marrow by the process of hematopoiesis. Their basic role in the body is defensive. They are responsible for the recognition, neutralisation, and destruction of various pathogens such as bacteria, viruses, parasites, and abnormal cells in the organism.

Hematopoiesis is a complicated process of blood cell formation from a pluripotent hematopoietic cell that, through the process of differentiation, passes into a unipotent stem cell dedicated to leukocytes from which mature forms of leukocytes are produced through several developmental stages, which are released into the peripheral blood. Basically, mature leukocytes are classified into two large groups:

- granulocytes (neutrophils, eosinophils and basophils),
- agranulocytes (lymphocytes and monocytes) [1] [2].

Immature forms of leukocytes, under physiological conditions, are not present in peripheral blood. Their presence is very significant in the diagnosis of various pathological conditions. The most common immature forms of leukocytes encountered in peripheral blood are blasts, promyelocytes, myelocytes, and metamyelocytes. The presence of immature forms of leukocytes indicates the existence of a pathological process in the body that leads to hyperfunction of the bone marrow, accelerated production of leukocytes and the release of immature forms of leukocytes into the peripheral blood.

In addition to immature forms of leukocytes, in the diagnosis of various diseases, the recognition of abnormal leukocytes is significant. The most common abnormalities encountered in practice relate, for example, to the existence of atypical lymphocytes that are identified in viral diseases, the presence of hyper-segmented neutrophils that are an accompanying symptom of megaloblastic anaemias, the presence of hyposegmented neutrophils that indicate a disorder in the maturation of granulocytes, the presence of plasma cells that are an accompanying symptom of multiple myeloma, etc. [3].

Leukocyte differentiation is one of the key diagnostic procedures in clinical medicine. The process itself represents a detailed analysis of different types of white blood cells and their percentage share in the total number of leukocytes. Changes in the total number of leukocytes, as well as changes in the percentage share of certain types of white blood cells, can be a significant diagnostic marker of various pathological conditions [4].

In all conditions, when there are any suspicions of a pathological process in the body within the regular complete blood count, a microscopic examination of the blood smear is performed, where the number and morphological characteristics of blood cells are analysed in detail. This review provides very significant initial data that will allow the clinician, together with patient history and any other symptoms that may be present, to select other methods to confirm the suspected diagnosis [5].

The reference method for the optical examination of blood smears is light microscopy, which ensures high-quality data. However, the large number of samples and the length of time it takes to prepare them, the lack of time in everyday work and the lack of educated staff are the most significant disadvantages of this method. Therefore, today, automatic analysers are increasingly present in haematology laboratories, which enable very precise and accurate differentiation of blood cells in the tested sample. For this purpose, the analyser used in the routine work in the haematology laboratory at the Clinical Center University of Sarajevo (UKC Sarajevo) - Department for Clinical Biochemistry and Laboratory Medicine is Sysmex XN-3100, manufactured by SYSMEX CORPORATION, Kobe, Japan.

## Materials and methods

Whole blood samples taken by venipuncture in vacuum containers with EDTA anticoagulant (BD Vacutainer, Becton Dickinson, United Kingdom) were used as study material. The data obtained from the analysis of samples were collected in the period January-March 2024. The inclusion of patients was based on routine clinical practice. These were patients suffering from anaemia, leukaemia or cancer for whom their doctor requested an optical microscopic examination of peripheral blood or the analyser performed an optical examination based on previously set criteria (flags). A total of 253 samples were analysed and classified according to the following criteria:

By gender: two groups - men (n = 121) and women (n = 132);

By age: four groups - children up to 18 years (n=22), young adults aged 19-40 (n = 21), middle- aged adults aged 41-60 (n = 65), and elderly patients over 61 years (n = 145);

By total leukocyte count: three groups - leukocytosis (leukocyte count >10x10/L; n = 88), leukopenia (leukocyte count <3x10/L; n = 53), and normal leukocyte count (3-10x10/L; n = 112).

The entire evaluation process was carried out with the approval of the Ethics Committee of the Clinical Center of the University of Sarajevo, respecting all the principles of the Declaration of Helsinki.

The analysis of all samples was made in the haematology laboratory at the UKC Sarajevo - Department for Clinical Biochemistry and Laboratory Medicine on the automatic analyser Sysmex XN-3100, manufactured by SYSMEX CORPORATION, Kobe, Japan. The results of the performed analyses were collected and statistically processed.

Sysmex XN-3100 is a modern, multifunctional, fully automated analyser whose use in everyday operation significantly shortens the time required for analysis and increases the number of samples that can be analysed per unit of time. Leukocyte analysis on this analyser is based on the results of two combined methods: fluorescent flow cytometry and light scattering method. Fluorescent flow cytometry uses fluorescent dyes that bind to nucleic acids in cells. Laser light excites these dyes, creating a fluorescent signal (SFL-Side Fluorescence Light) that is detected and used for cell analysis. The fluorescence intensity depends on the amount of nucleic acids inside the cell [6]. The light scattering method measures the intensity of transmitted light passing through cells, providing information on their morphological characteristics. The scattering measurement is performed at a small angle (between 0°C and 5°C) or at a right angle in relation to the direction of the laser beam. In doing so, two parameters are detected: Forward Scatter (FSC), which is a measure of the size of the cells, and Side Scatter (SSC), which is a measure of the granularity and internal structure of the cells [7].

By combining these three parameters, the results of the performed analysis are obtained, which can be auto-validated by the analyser (when the results are within the reference limits and there are no detected abnormalities), or the analyser gives a recommendation for a microscopic examination of the blood smear and mark that sample with a flag [6]. In this case, the analyser can automatically prepare a blood sample smear using SP-50 (Slide preparation) and DI-60 (Digital Imaging).

SP-50 is a part of the analyser that automatically prepares a high-quality blood smear. The amount of sample, the angle and speed of the smear are determined by the hematocrit. Blood smear staining was performed using the standard MGG (May-Grünvald-Giemsa) procedure. The dried blood smear was fixed with May-Grünvald solution (duration 2 min and 24 s). Fixation continued in diluted May-Grünvald solution with phosphate buffer in a ratio of 1:10 for 48 seconds. Further staining continued with diluted Giemsa solution and phosphate buffer in a ratio of 1:25 (duration 5 min and 12 s). After staining, a 1-minute rinse was performed with phosphate buffer, followed by drying the preparation for 3 min and 36 s. The total time required for blood smear staining was 13 minutes. Further, the process involves the DI-60 part of the analyser, which uses a motorised light microscope with LED illumination and a digital camera. In this part, the blood smear is scanned, and at least 110 cells are detected, which are photographed and further analysed using the CellVision software. The software analyses each individual cell and classifies it into appropriate groups using pre-defined algorithms [7] [8]. The data obtained in this way are primary results (preclassification) which are further analysed by laboratory staff with an adequate level of education, where they can confirm or correct them, thus creating secondary data (reclassification). Only data processed in this way are recorded and verified [8].

Statistical analysis was done in MedCalc (Version 20.218-64-bit, Ostend, Belgium). The comparison was made on the basis of the guidelines given in the CLSI (Clinical & Laboratory Standards Institute) guide [9].

The degree of agreement among the same parameters analysed on the Sysmex XN-3100 (preclassification and reclassification) was estimated based on a non-parametric Passing-Bablok regression analysis. This analysis is used to evaluate the agreement between the two measurement methods. The statistically significant value for each analysis is p<0.05. The regression equation is represented as y=ax+b in which the proportional difference between the two analyses is represented by a slope (b) and the constant difference by an intercept (a) of the regression line. The confidence interval (95% CI) explains whether the value of a is different from zero and whether the value of b is different from 1 or not. If CI includes zero for value a, it can be concluded that there is no constant bias between the two measurements. At the same time, if CI includes 1 it can be concluded that there is no proportional bias between the two measurements. Then y=x and methods can be used interchangeably. Otherwise, certain corrections must be applied in cases where the values of 1 for slope and 0 for intercept are not covered by a confidence interval, indicating a significant deviation [10] [11].

Further statistical analysis includes the Bland-Altman plot. This analysis is used to estimate the agreement between two measurements or methods of measuring the same parameter. It gives an instrumental graphical assessment of the consistency of the two methods. The key data for the interpretation of this analysis undermine the average difference (bias) between the values given by the two measurements. This parameter shows how different the tested methods are, and in ideal conditions, the bias should be as close as possible to zero, thus minimising the difference between the methods. With this method, we also get data related to the stacking limits. They are defined as bias ±1.96 x standard deviation and actually represent the limits within which the results of 95% of the conducted analyses should be. All data obtained by this analysis are presented graphically. On the y-axis, the value of the difference between the measurements is applied, and on the x-axis, the mean value of the measurement is used. From the graph, differences in the distribution of data can be observed, which can indicate the variability of the method in different ranges of measurements [12] [13].

## Results

**Figure 1:**
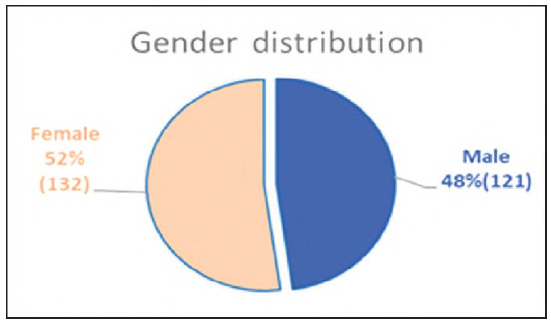
Gender structure of the analysed samples.

**Figure 2:**
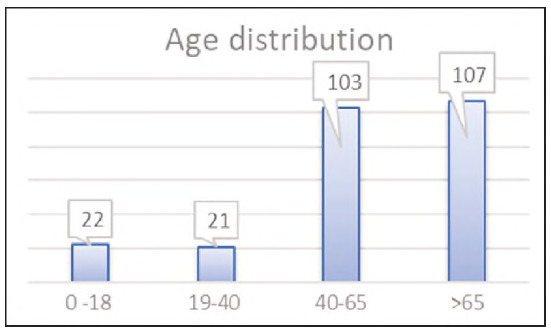
Age structure of analysed samples.

**Figure 3:**
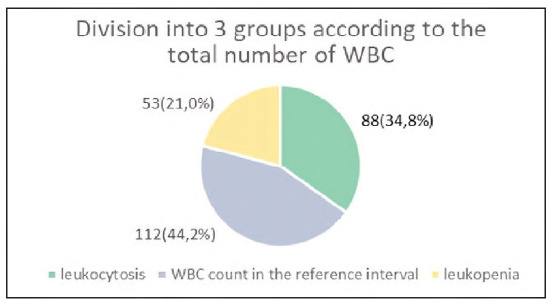
Percentage of groups based on the number of WBCs in the total number of samples.

The analysis was performed on 253 samples of gender and age structure, shown in Figure 1 and Figure 2. The samples were divided on the basis of the total number of leukocytes into 3 groups: leukopenia, leukocytosis and samples with the number of leukocytes in the reference interval. The percentage representation of these groups in the total number of analysed samples is shown in Figure 3.

All samples included in the analysis were analysed on the Sysmex XN-3100 analyser, where digital optical microscopy was performed. The obtained data were collected immediately after automatic microscopic differentiation and represent a reclassification. All samples were further checked by a medical biochemistry specialist who confirmed or corrected the differentiated cells based on the available colour photo of the analysed cells before final verification. In this way, the data that make up the reclassified data were collected.

The collected data were analysed by Passing-Bablok regression and Bland-Altman plot analysis, which compared the data obtained by preclassification (P) and the data obtained after reclassification (R), as two methods. The following cells were analysed: segmented neutrophils, basophils, eosinophils, lymphocytes, monocytes, blasts and total immature granulocytes (bands, metamyelocytes, myelocytes and promyelocytes). The data obtained are shown in Table 1.

**Table 1:** Results of Passing-Bablok regression and Bland-Altman plot analysis.

	SEG% P	SEG% R	BASO% P	BASO% R	EOS% P	EOS% R	LYM% P	LYM% R	MONO% P	MONO% R	IG% P	IG% R	BLAST% P	BLAST% R
Sample size	253													
Lowest value	0	0	0	0	0	0	0	1	0	0	0	0	0	0
Highest value	92	92,5	63,6	50	50	37,5	100	100	86	78,2	39,2	41	45,9	41,8
Arithmetic mean	49,81	51,4933	0,9945	0,8704	1,7719	1,5107	29,849	31,7063	7,3909	7,298	6,581	6,036	1,34	0,9198
Median	52,5	55,5	0	0	0,5	0,5	24	24	5,6	5,5	4	4	0	0
Standard deviation	25,063	24,6885	5,6679	4,213	4,5472	3,5679	23,246	24,7145	9,1465	8,7872	7,077	6,1568	6,728	4,691
Standard error of the mean	1,5757	1,5522	0,3563	0,2649	0,2859	0,2243	1,4615	1,5538	0,575	0,5524	0,445	0,3871	0,423	0,2949
**Passing-Bablok regression**														
**Systematic differences**														
Intercept A	0,6000		0,0000		0,0000		-0,0257		0,0000		0,1558		0,0000	
95% Cl	0,09567 to 1,3394		0,0000 to 0,0000		0,0000 to 0,0000		0,7494 to-0,1933		0,0000 to 0,007463		0,0000 to 0,4359		0,0000 to 0,0000	
**Proportional differences**														
Slope В	1,0000		1,0000		1,0000		1,0200		1,0000		0,8844		1,0000	
95% Cl	0,9879 to 1,0108		1,0000 to 1,0000		1,0000 to 1,0000		1,0096 to 1,0333		0,9963 to 1,0000		0,8205 to 0,9545		0,9639 to 1,4737	
**Random differences**														
Residual Standard Deviation<br>(RSD)	4,9031		1,1071		1,7847		3,7358		0,9995		2,52		2,4299	
± 1.96 RSD Interval	-9,6101 to 9,6101		2,1699 to-2,1699		3,4980 to-3,4980		7,3221 to-7,3221		1,9590 to-1,9590		4,9391 to-4,9391		4,7626 to-4,7626	
**Linear model validity**														
Cusum test for linearity	No significant<br>deviation from<br>linearity (P=0,22)		No significant<br>deviation from<br>linearity (P=0,62)		No significant<br>deviation from<br>linearity (P=0,87)		No significant<br>deviation from<br>linearity (P=0,50)		No significant<br>deviation from<br>linearity (P=0,87)		Significant<br>deviation from<br>linearity (P<0,01)		Significant deviation<br>from linearity<br>(P<0,01)	
**Spearman rank correlation coefficient**														
Correlation coefficient	0,963		0,892		0,870		0,986		0,947		0,789		0.711	
Significance level	P<0,0001		P<0,0001		P<0,0001		P<0,0001		P<0,0001		P<0,0001		P<0,0001	
95% Cl	0,953 to 0,971		0,863 to 0,914		0,837 to 0,897		0.982 to 0,989		0,933 to 0,959		0,737 to 0,831		0.644 to 0,767	
**Bland-Altman plot**														
Arithmetic mean	1,6838		-0,1241		-0,2613		1,8573		-0,09289		-0,5451		-0,4202	
95% Confidence interval	0,8376 to 2,5300		-0,3170 to 0,06875		-0,5715 to 0,04893		1,2099 to 2,5047		-0,2672 to 0,08140		-1,0013 to-0,08883		-0,8416 to 0,001263	
P (H_o_: Mean=0)	0,0001		0,2062		0,0984		<0,0001		0,2949		0,0194		0,0507	
Lower limit	-11,712		-3,1770		-5,1716		-8,3915		-2,8518		-7,7671		-7,0912	
95% Confidence interval	13,1601 to-10,263		-3,5070 to-2,8470		-5,7024 to-4,6408		-9,4994 to-7,2835		-3,1500 to-2,5535		-8,5478 to-6,9864		-7,8124 to-6,3700	
Upper limit	15,0796		2,9288		4,6491		12,1061		2,6660		6,6770		6,2509	
95% Confidence interval	13,6314 to 16,5277		2,5988 to 3,2588		4,1182 to 5,1799		10,9982 to 13,2140		2,3677 to 2,9642		5,8963 to 7,4577		5,5297 to 6,9721	

Based on the results of the Passing-Bablok regression, proportional and constant bias can be inferred within the analysed groups. Some parameters show satisfactory agreement, while some show constant and/or proportional bias.

A constant bias indicates that a particular method yields values higher or lower than another for a constant value, while a proportional bias means that one method yields values higher or lower than another for a value proportional to the size of the measured parameter [14]. In the group of segmented neutrophils, there is a small constant bias that can be observed from the value of the intercept (a) that does not include a value of 0 within 95% CI. In the group of lymphocytes and immature granulocytes, a proportional bias is present that is observed based on a slope value (b) that does not include a value of 1 within 95% CI.

The linearity of the methods is satisfactory for all analysed parameters except blasts and immature granulocytes, where there is a statistically significant deviation from linearity. Therefore, for these two data groups, Passing-Bablok regression cannot be applied.

Spearman's correlation coefficient for parameters with satisfied linearity shows a value greater than 0.70, indicating a powerful correlation between the methods [15].

A graphical representation of the Passing-Bablok regression analysis for all analysed cells is presented in Figure 4, Figure 5, Figure 6, Figure 7, Figure 8, Figure 9, Figure 10, Figure 11. The conducted Bland-Altman plot analysis shows the following results (Figure 11, Figure 12, Figure 13, Figure 14, Figure 15, Figure 16, Figure 17).

**Figure 4:**
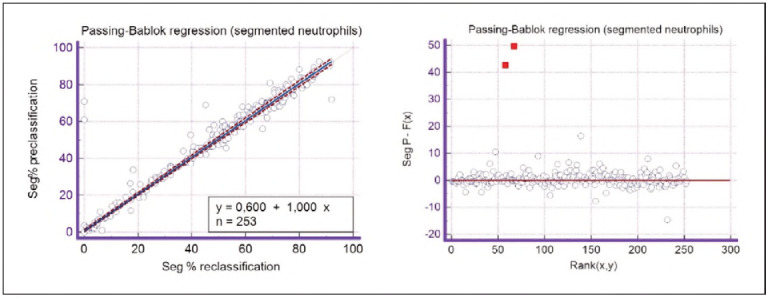
Passing-Bablok regression analysis for segmented neutrophils.

**Figure 5:**
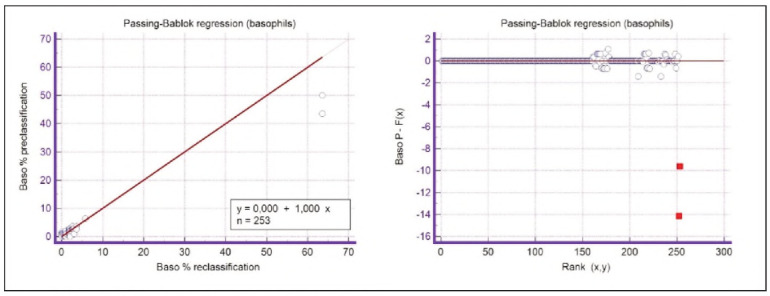
Passing-Bablok regression analysis for basophils.

**Figure 6:**
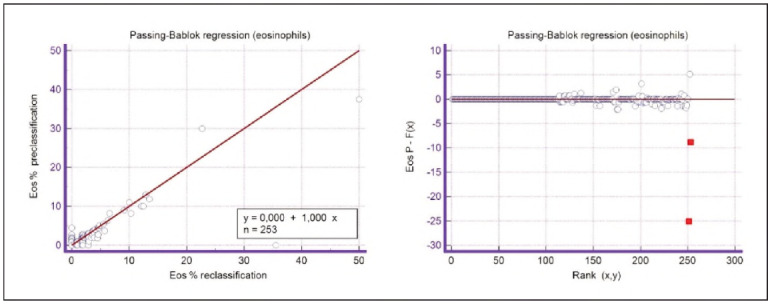
Passing-Bablok regression analysis for eosinophils.

**Figure 7:**
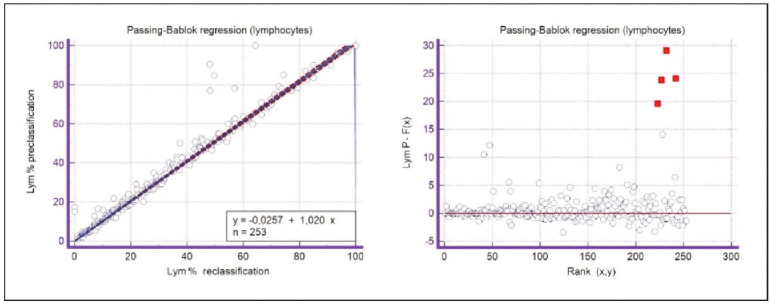
Passing-Bablok regression analysis for lymphocyte.

**Figure 8:**
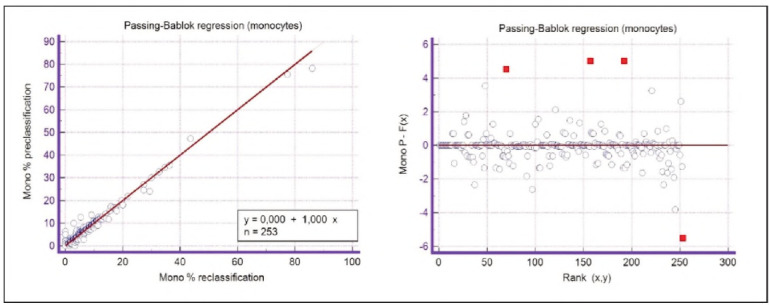
Passing-Bablok regression analysis for monocytes.

**Figure 9:**
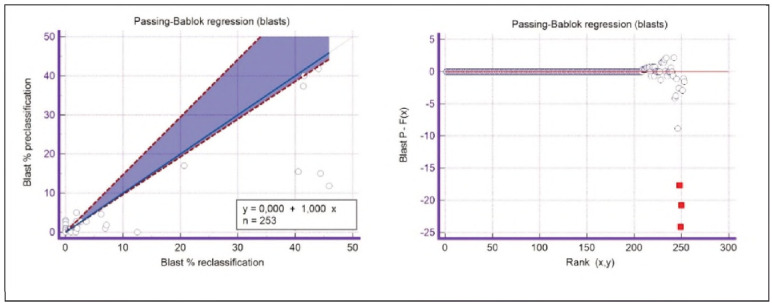
Passing-Bablok regression analysis for blasts.

**Figure 10:**
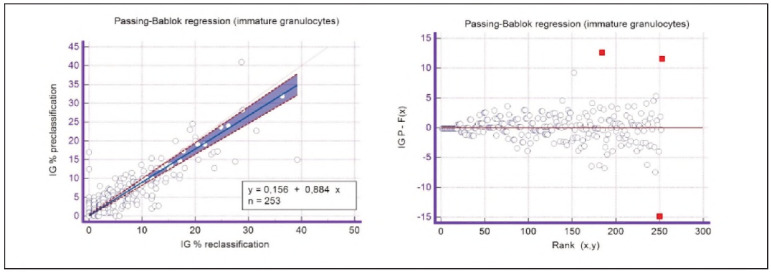
Passing-Bablok regression analysis for immature granulocytes.

**Figure 11:**
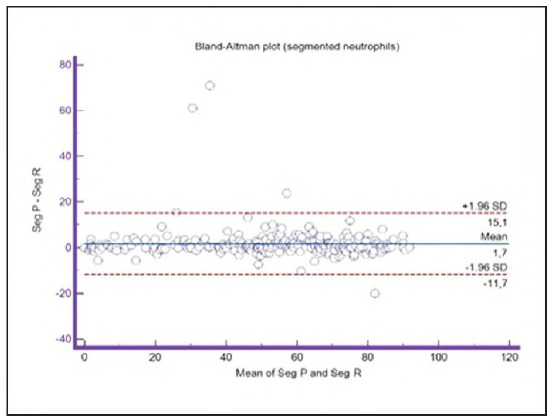
Bland-Altman plot for segmented neutrophils.

**Figure 12:**
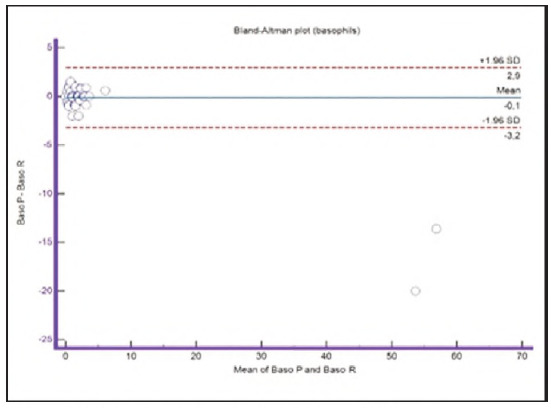
Bland-Altman plot for basophils.

**Figure 13:**
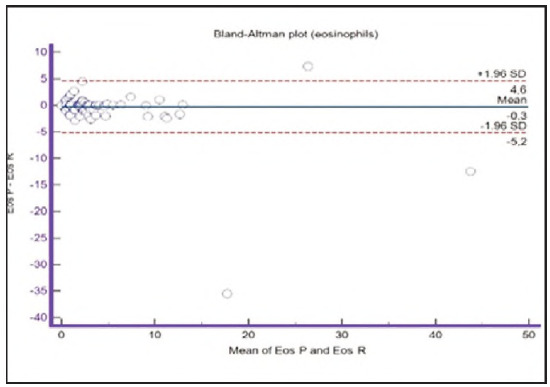
Bland-Altman plot for eosinophils.

**Figure 14:**
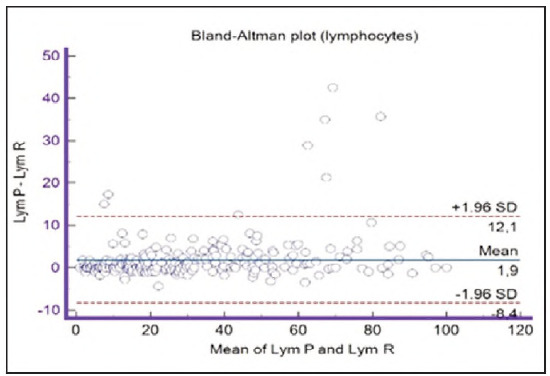
Bland-Altman plot for lymphocytes.

**Figure 15:**
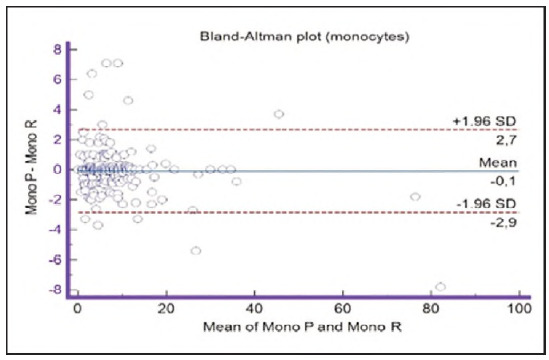
Bland-Altman plot for monocytes.

**Figure 16:**
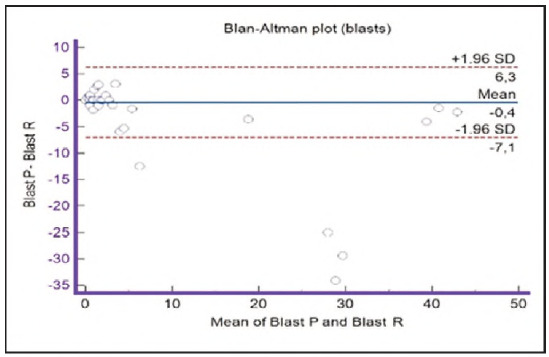
Bland-Altman plot for blasts.

**Figure 17:**
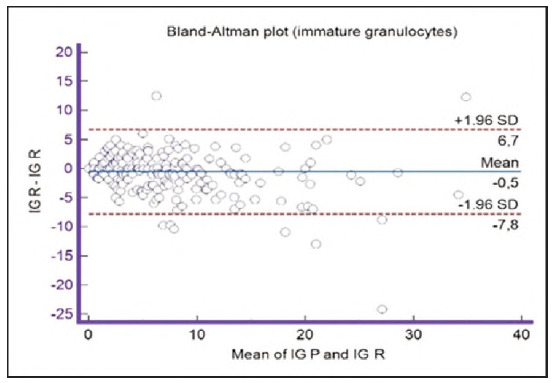
Bland-Altman plot for immature granulocytes.

## Discussion

Based on the presented results of the Passing-Bablok regression analysis, satisfactory linearity can be observed in all groups of analysed cells except for blasts and immature granulocytes. Since linearity in these groups is not met, further Passing-Bablok regression analysis for these groups cannot be applied.

Based on the intercept values, a consistent slight bias was observed only in the group of segmented neutrophils, indicating that the primary classification consistently reported higher segmented neutrophil counts compared to the reclassification across the entire range of analysis. In contrast, the intercept values for the other analysed cell groups were within acceptable limits and did not indicate any systematic bias.

By analysing the slope value, which is a measure of the proportional bias, a small deviation can be observed in lymphocytes, which should be especially taken into account in lymphocytosis, where a more considerable bias is expected to be proportional. By examining the data, it was noticed that the preclassification gives a higher number of lymphocytes compared to the reclassification. Proportional bias was also observed in the group of immature granulocytes, but due to the absence of linearity, this parameter is not interpreted in this way.

Correlation within the analysed groups was examined by determining the Spearman coefficient. The obtained data in all groups show a value above 0.7, which is an indicator of the existence of a very strong correlation. The highest value of Spearman's coefficient was observed in the lymphocyte group (r=0.986) and the lowest in the eosinophil group (r=0.870).

Further statistical analysis was conducted through the Bland-Altman plot analysis, and the results obtained show:

- Segmented granulocytes: the mean difference is 1.68 (0.84-2.53), with 5 points outside the agreement area - limits of agreement (mean ± 1.96SD). In their research, Kim et al. [16] also obtained a positive value of mean difference (0.28), which correlates with our results. That means that, on average, preclassification gives a higher value compared to reclassification. On the other hand, Yoon et al. [17] obtained a negative value of the mean difference (-1.30), as opposed to our results. Still, considering that it is only made on samples with leukopenia, we should take this comparison with a slight reserve.
- Basophils: the mean difference is -0.12 (-0.32-0.07), with 2 points significantly outside the agreement area - out of the limits of agreement (mean ± 1.96SD). These two deviations were observed in severe basophilia of the samples. As opposed to our results, Kim et al. [16] obtained a positive mean value (0.64), which means that their preclassification gave higher values on average compared to the reclassification, and ours gave lower values. Yoon et al. [17] also obtained a positive mean difference value (2.55) but with a significantly higher value, which may be significant for basophils, which are, in general, presented in a lower percentage in peripheral blood.
- Eosinophils: the mean difference is -0.26 (-0.57-0.05), with 3 points outside the agreement area - out of the limits of agreement (mean ± 1.96SD). A particularly significant deviation was observed in 2 samples with eosinophilia. Kim et al. [16] also obtained a negative mean difference value (-1.40), as did Yoon et al. [17], whose value is -1.68.
- Lymphocytes: the mean difference is 1.86 (1.21-2.50), with 8 points outside the agreement area - out of the limits of agreement (mean ± 1.96SD). A particularly significant deviation was observed in 5 samples with lymphocytosis. Kim et al. [16] also obtained a positive mean difference value (2.17), as did Yoon et al. [17], whose value is 0.08.
- Monocytes: the mean difference is -0.09 (0.27-0.08), with 12 points outside the agreement area - out of the limits of agreement (mean ± 1.96SD). Kim et al. [16] also obtained a negative mean difference value (-2.50) but with a significantly higher value [16]. Yoon et al. [17] obtained a negative value too (-0.30).
- Blasts: the mean difference is -0.42 (-0.84-0.00), with 4 points outside the agreement area out of the limits of agreement (mean ± 1.96SD). A particularly significant deviation was observed in 3 samples in which a significantly higher number of blasts was determined by reclassification compared to preclassification. A detailed analysis of these 3 samples showed that the reduced number of blasts in the preclassification was the result of a falsely increased number of lymphocytes. The reclassification performed corrected this result and primarily classified a certain number of lymphocytes reclassified into blasts. Kim et al. [16] obtained a positive mean difference value (0.06), as did Yoon et al. [17], whose value is 0.62.
- Immature granulocytes: the mean difference is -0.54 (-1.00--0.09), with 9 points outside the agreement area -out of the limits of agreement (mean ± 1.96 SD). There is a visible dispersion of data around the mean, and on all samples outside the agreement area, we notice a significant deviation. Detailed analysis of the points outside the mean showed that the most common replacement in the group of immature granulocytes occurs with segmented neutrophils. As opposed to our results, Kim et al. [16] obtained a positive mean value (0.74), the same as Yoon et al. [17], whose value is 1.39.

## Conclusion

Based on the analysis performed, the Sysmex XN-3100 analyser shows very high performance in leukocyte analysis and their differentiation using digital microscopy. The preclassification, as the primary result given by the Sysmex XN-3100 analyser, in relation to the reclassification performed by trained laboratory professionals, is satisfactory for all groups of leukocyte cells. For a certain number of samples in which blasts and immature granulocytes are detected, additional attention should be paid to the reclassification, or the result should be checked by optical microscopy. The advantage of Sysmex XN-3100 is that samples containing these cells are flagged by the analyser, which directs laboratory professionals to samples that require additional laboratory processing.

## Dodatak

### List of abbreviations

CI, confidence interval;<br>CLSI, clinical & laboratory standards institute;<br>DI, digital imaging;<br>FSC, forward scatter;<br>NRBC, nucleated red blood cells;<br>P preclassification;<br>R, reclassification;<br>SFL, side fluorescence light;<br>SP slide preparation;<br>SSC, side scatter;<br>UKC, Clinical Center University of Sarajevo.

### Ethics approval

The Ethics Committee of The Clinical Center University of Sarajevo approved the present study. All procedures were performed in accordance with the ethical standards of the Institutional Review Board and The Declaration of Helsinki.

### Authors' contribution

Nermina Klapuh-Bukvić designed the research study. All authors performed the research. Zehra Kurtanović and Damir Seper analysed the data. All authors wrote the manuscript. Nermina Klapuh- Bukvić reviewed and edited the manuscript. All authors contributed to editorial changes in the manuscript. All authors read and approved the final manuscript.

### Acknowledgements

Not applicable.

### Conflict of interest statement

All the authors declare that they have no conflict of interest in this work.
